# Comparative analysis of laparoscopic versus open surgery in appendicitis management

**DOI:** 10.6026/973206300220197

**Published:** 2026-01-31

**Authors:** Ashish Kumar Shukla, Kusum Mahour, Pradeep Rathore

**Affiliations:** 1 Department of General Surgery, Government Medical College, Datia, Madhya Pradesh, India

**Keywords:** Appendicitis, laparoscopic appendectomy, open appendectomy, surgical site infection, length of stay, minimally invasive surgery

## Abstract

The acute appendicitis is still a common surgical emergency, but the most effective intervention between the traditional open appendectomy (OA) and laparoscopic appendectomy (LA) is still a controversial topic. This was a retrospective cohort study on 250 patients who were treated between March 2023 and March 2025 in tertiary care of Datia, Madhya Pradesh, with operations involving LA (n=155) and OA (n=95) and compared between them the operation time, hospitalization period, and cases of postoperative complications. LA was characterized by a greater length of operation but much shorter hospital stay, reduction of surgical-site infections and rapid functional recovery. The 24 hours pain scores were lower in the LA group. LA was more effective in terms of postoperative results, when treating the majority of acute appendicitis cases.

## Background:

The most frequent example of acute abdominal pain that leads to surgical treatment is acute appendicitis, the risk of which is about 7-8% in a lifetime [1]. Since its original description by Charles McBurney, open appendectomy (OA) remains the gold-standard therapy, and shows outstanding effectiveness in the prevention of life-threatening complications of perforation and sepsis [2]. The process is quite developed, technically simple, and quite efficient. The topography of appendicitis treatment started to change with the introduction of minimal invasive surgery. The first laparoscopic appendectomy (LA) was done by Kurt Semm in 1982 and since then the method has gained popularity and is widely accepted [3]. LA has the theoretical benefits of minimally invasive techniques, which are low postoperative pain, better cosmesis, and possible faster recovery and a return to daily activities [4]. In addition, the panoramic vision of the abdomen provided by the laparoscope may be especially beneficial in cases of difficult diagnostics, in particular, in the female patient of age of childbearing when the gynaecological pathology can confuse with appendicitis [5]. The last 30 years have witnessed several randomized controlled trials and meta-analyses that have compared the results of LA and OA. A classic Cochrane review, and updates thereafter, have been able to demonstrate constantly that LA has a much lower incidence of surgical site infections (SSIs), less postoperative pain, and a reduced length of hospital care than OA [6, 7]. Nevertheless, the same analyses had in the past also cited that LA correlated to a longer operative period and that it was possible that there were an increased number of intra- abdominal abscesses (IAAs) [8]. Specifically, this latter issue moderated the original interest in a total shift to laparoscopic-first, particularly in situations of complicated (gangrenous or perforated) appendicitis. Regardless of the abundance of the existing literature, there is a research gap. Initial research was marred by high learning curve of laparoscopy and operative procedures, such as how to irrigate and retrieve the specimen, have since developed [9]. The evaluation of such procedures in the context of particular institutions has to be constant in order to mirror the current practice, local experience, and patient groups. Assessing performance within the real world setting gives useful data concerning quality control, protocol development, and evidence-based decision making. Therefore, it is of interest to conduct a retrospective comparative analysis of clinical outcomes between laparoscopic and open appendectomy in a contemporary institutional cohort with acute appendicitis, evaluating postoperative recovery, morbidity, SSIs, IAAs, and operative efficiency to inform current evidence-based practice.

## Materials and Methods:

## Study design and patient population:

A total of 250 patients met the eligibility criteria and were included in the final analysis from a period between March 2023 to March 2025 in tertiary care hospital of Datia, M.P. Patients were divided into two groups based on the surgical approach they received: the Laparoscopic Appendectomy (LA) group and the Open Appendectomy (OA) group. The choice of surgical procedure was based on the discretion of the attending surgeon, taking into account patient factors and surgeon preference.

## Inclusion and exclusion criteria:

Inclusion criteria were: (1) age 16 years or older; (2) a clinical and/or radiological diagnosis of acute appendicitis; and (3) undergoing either LA or OA. Exclusion criteria were: (1) patients who underwent an incidental appendectomy during another procedure; (2) age < 16 years; (3) patients with a preoperative diagnosis of an appendiceal mass or malignancy; (4) pregnant patients; and (5) cases with incomplete or missing follow-up data.

## Data collection:

A standardized data collection form was used to extract information from the hospital's electronic health records and surgical database. The collected data included:

[1] Demographics: Age, gender, and Body Mass Index (BMI).

[2] Clinical Data: Preoperative leukocyte count and Alvarado score.

[3] Intraoperative Data: Surgical approach (LA or OA), operative time (from skin incision to skin closure), conversion from LA to OA, and intraoperative findings (e.g., simple, gangrenous, or perforated appendicitis).

[4] Postoperative Outcomes: Length of hospital stay (LOS), postoperative pain score on a 10-point Visual Analog Scale (VAS) at 24 hours, time to return to normal activities (as documented in follow-up notes), and 30-day postoperative complications. Complications were classified as SSI (superficial or deep), IAA, postoperative ileus requiring intervention, and hospital readmission.

## Surgical procedures:

[1] Laparoscopic Appendectomy (LA): A standard three-port technique was used. A 10-mm umbilical port was established for the camera, and two 5-mm working ports were placed in the left lower quadrant and suprapubic region. The mesoappendix was divided using either an electrocautery device or a vessel-sealing device. The appendix base was secured with endoscopic loops or a surgical stapler. The specimen was removed in an endoscopic retrieval bag.

[2] Open Appendectomy (OA): The procedure was typically performed through a McBurney or Rocky-Davis incision in the right lower quadrant. The appendix was identified, the mesoappendix was ligated and divided, and the appendix base was ligated and transected.

## Statistical analysis:

All statistical analyses were conducted using SPSS Statistics Version 28.0 (IBM Corp., Armonk, NY). Continuous variables were expressed as mean ± standard deviation (SD) and were compared using the independent samples t-test or Mann-Whitney U test where appropriate. Categorical variables were presented as frequencies and percentages (%) and were compared using the Chi-square test or Fisher's exact test. A p-value of <0.05 was considered to be statistically significant.

## Results:

A total of 250 patients were included in the study. Of these, 155 (62.0%) underwent LA and 95 (38.0%) underwent OA. Ten patients in the LA group (6.5%) required conversion to an open procedure due to dense adhesions or difficult anatomy; these patients were analyzed in the OA group as per an intention-to-treat principle for outcome analysis, but their initial approach was noted. The baseline demographic and clinical characteristics were similar between the two groups, with no statistically significant differences in age, gender, BMI, or preoperative Alvarado scores, as detailed in Table 1. The proportion of complicated appendicitis (gangrenous or perforated) was also comparable between the two groups. The primary intraoperative and postoperative outcomes are summarized in Table 2. The mean operative time was significantly longer for LA than for OA (65.2 ± 15.1 min vs. 55.8 ± 12.5 min; p<0.001). Conversely, the LA group demonstrated significantly better postoperative recovery metrics. The mean LOS for LA patients was approximately half that of OA patients (2.1 ± 0.8 days vs. 4.3 ± 1.5 days; p<0.001). Postoperative pain, as measured by the VAS score at 24 hours, was significantly lower in the LA group. Consequently, the time to return to normal activities was also significantly shorter for patients who underwent LA. The rates of 30-day postoperative complications are presented in Table 3. The overall complication rate was lower in the LA group. The incidence of SSI was significantly lower in patients undergoing LA (3.2%) compared to those undergoing OA (9.5%) (p=0.035). There was no statistically significant difference in the rates of IAA, postoperative ileus, or 30-day hospital readmission between the two groups.

## Discussion:

This comparative trial attests that laparoscopic appendectomy has significant clinical benefits to the conventional open method in the treatment of acute appendicitis. We find out that a marginally longer operation period may be necessary in LA, but at the expense of much less postoperative pain, a divided hospitalization, a lower wound infection rate, and a quicker recovery period. These findings are consistent with an extensive amount of research that has been developed throughout the last years and support the argument in favor of LA as the modality of surgery [7, 10]. The greatest advantages as noted in our LA cohort were associated with the postoperative recovery. The average length of stay was decreased by two complete days and the period when they go back to regular activities was decreased by half. It has far-reaching implications on the issues of patient comfort, patient satisfaction, healthcare economics, and productivity in the society [11]. The shortened hospital stay saves healthcare costs while enabling faster return to work and normal activities, benefits confirmed by recent prospective studies showing laparoscopic appendicectomy's superiority in pain reduction (VAS 3.2 vs 5.1), quicker recovery, and fewer complications like urinary retention [12, 13]. These advantages stem directly from LA's minimally invasive nature, featuring fewer incisions, reduced tissue trauma, and lower systemic inflammation. We have discovered that the incidence of SSIs in the LA group was much lower (3.2% vs. 9.5%). This is among the most frequently reported benefits of LA in the literature [6, 14]. The lower port-site cuts, the less exposure of the peritoneal space to the outside world, and the minimal direct contact with tissues are all contributing factors. The relevance of this discovery is more so considering the fact that SSIs represent a significant cause of postoperative morbidity, resulting in lengthy hospitalization, escalated expenses, and unfavorable outcomes in patients. Similar findings were reported in a prospective comparative study of 100 patients, where laparoscopic appendicectomy showed SSI rates of 4% versus 12% for open appendicectomy (p=0.04), alongside shorter hospital stays (2.3 vs 4.1 days) [15].

Another interesting observation during our analysis was that there was no statistically significant difference in intra-abdominal abscesses rate between the two groups. Increased risk of IAA was a significant issue with LA in the past as it was usually because of the failure to perform adequate lavage to the peritoneal cavity or because of contamination during the extraction of a specimen into the peritoneal cavity [8]. Our data, however, in agreement with more recent meta-analyses, indicate that this risk has been mitigated to a great extent [16]. This equalization of risk has probably been brought about by the development of laparoscopic procedure, such as the regular use of specimen retrieval bags to avoid spillage and the use of more effective irrigation and suction devices [17, 18]. This renders null one of the final significant arguments against the routine use of LA, even in the case of perforated appendicitis. The single parameter on which OA occurred superiorly was operating time. This is because our result of an average operative time that is about 10 minutes longer in LA is congruent with numerous published series [19]. The reason behind this difference can be explained by the fact that pneumoperitoneum, equipment set-up, and the time of dissection may be longer in complicated cases. This slight increment in time spent at the operating room, however, is outweighed by the days saved in the recovery period as shown by the much improved postoperative outcomes. There are several limitations of this study with regard to its design. Being a retrospective study, it is prone to selection bias because the decision of the type of surgery was left at the will of the surgeon. In some cases, it is probably possible that more complicated cases or patients with major comorbid conditions were selectively not based on an open approach, but our baseline data indicated that groups were reasonably comparable. Also, the research was carried out in one center, and this might not be applicable in other environments with dissimilar resources or surgeon experience. Lastly, the long-term outcomes, including incisional herniaity and adhesive small bowel obstruction were not evaluated.

## Conclusion:

This comparative analysis of 250 patients shows that laparoscopic appendectomy is a safe and most effective therapy of acute appendicitis, which has clear and significant benefits over open appendectomy. The patients who have LA enjoy reduced pain, significantly reduced hospital stay and reduced chances of wound infection, and a significantly reduced time to resume normal functions. These advantages make laparoscopic appendectomy a standard of care and most desirable surgical mode to use on the greater part of the patients who present with acute appendicitis during the contemporary surgical age.

## Figures and Tables

**Table 1:** Demographic and baseline clinical characteristics

**Characteristic**	**LA Group (n=155)**	**OA Group (n=95)**	**p-value**
Mean Age (years) ± SD	35.8 ± 14.1	37.2 ± 15.5	0.48
Gender (Male), n (%)	91 (58.7%)	53 (55.8%)	0.65
BMI (kg/m^2^), mean ± SD	26.1 ± 4.5	26.8 ± 4.9	0.29
Alvarado Score, mean ± SD	7.2 ± 1.5	7.4 ± 1.3	0.31
Complicated Appendicitis, n (%)	38 (24.5%)	26 (27.4%)	0.63

**Table 2:** Comparison of intraoperative and postoperative outcomes

**Outcome**	**LA Group (n=155)**	**OA Group (n=95)**	**p-value**
Operative Time (min), mean ± SD	65.2 ± 15.1	55.8 ± 12.5	<0.001
Length of Stay (days), mean ± SD	2.1 ± 0.8	4.3 ± 1.5	<0.001
Pain Score (VAS at 24h), mean ± SD	3.5 ± 1.2	5.8 ± 1.4	<0.001
Return to Normal Activities (days), mean ± SD	9.4 ± 3.1	18.2 ± 4.5	<0.001

**Table 3:** Comparison of 30-day postoperative complications

**Complication**	**LA Group (n=155)**	**OA Group (n=95)**	**p-value**
Surgical Site Infection (SSI), n (%)	5 (3.2%)	9 (9.5%)	0.035
Intra-abdominal Abscess (IAA), n (%)	4 (2.6%)	1 (1.1%)	0.48
Postoperative Ileus, n (%)	6 (3.9%)	5 (5.3%)	0.61
30-Day Readmission, n (%)	3 (1.9%)	2 (2.1%)	0.91
